# The *ADRB3* rs4994 polymorphism increases risk of childhood and adolescent overweight/obesity for East Asia’s population: an evidence-based meta-analysis

**DOI:** 10.1080/21623945.2020.1722549

**Published:** 2020-02-02

**Authors:** Chenyao Xie, Wenxi Hua, Yuening Zhao, Jingwen Rui, Jiarong Feng, Yanjie Chen, Yu Liu, Jingjing Liu, Xiaoqin Yang, Xiaojing Xu

**Affiliations:** a Department of Bioinformatics, School of Biology and Basic Medical Science, Soochow University, Suzhou, Jiangsu Province, China; b Medical School, Soochow University, Suzhou, Jiangsu Province, China; c Department of Biochemistry and Molecular Biology, School of Biology and Basic Medical Science, Soochow University, Suzhou, Jiangsu Province, China; d Department of Cell Biology, School of Biology and Basic Medical Science, Soochow University, Suzhou, Jiangsu Province, China

**Keywords:** ADRB3, genetic polymorphism, obesity, meta-analysis

## Abstract

Whether the Adrenoceptor Beta 3 (*ADRB3*) gene rs4994 polymorphism could affect the individual risk of childhood and adolescent overweight/obesity remains controversial. This meta-analysis was performed to estimate the prevalence of this polymorphism in overweight/obesity, and test the potential association by summarizing existing evidence. Comprehensive literature search in PubMed, Web of Science, Cochrane Library, Wanfang, and CNKI databases was performed to identify eligible data sets. Finally, 16 studies involving 5,147 overweight/obese cases and 7,350 non-obese controls were included for further synthetic analyses. Odds ratio (OR) and its corresponding 95% confidence intervals (CIs) were statistically calculated. Totally, 69.9% of the included subjects came from East Asia. In the meta-analysis for overall population, statistically significant associations with increased risk of childhood and adolescent overweight/obesity were identified in allele model (OR 1.23, 95% CI 1.10–1.38), heterozygote model (OR 1.39, 95% CI 1.16–1.68), and dominant model (OR 1.31, 95% CI 1.12–1.54). Further stratified analysis according to geographical regions revealed that the statistical significance could only be detected in the East Asia subgroup in allele model, homozygote model, heterozygote model, and dominant model. In summary, our meta-analysis indicated that the *ADRB3* rs4994 polymorphism could significantly increase the risk of childhood and adolescent overweight/obesity, especially for the East Asia’s population.

## Introduction

Emerging as a serious health problem worldwide, childhood and adolescent obesity, a complex and multifactorial metabolic disorder, could lead to not only improper physical and mental development [1–3], but also increased risk of medical complications like cardiovascular disease, dyslipidemia, asthma exacerbation, and metabolic syndrome [4]. The reciprocity between individual factors, including genetic variation like single nucleotide polymorphism (SNP), and lifestyle/environmental variables such as nutrition overbalance, lack of physical activity, and sedentary habit could interpret the variability in obesity predisposition between individuals in a given population.

Beta-adrenergic receptors, a subgroup of G-protein-coupled receptors, are involved in the regulation of energy expenditure [5]. As a member of this receptor family, the Adrenoceptor Beta 3 (ADRB3) gene, locating at 8p11.23 region of human genome, modulates catecholamine-induced stimulation of adenylate cyclase via the action of G proteins. This receptor expresses predominantly in adipocytes and functions in mediating lipolysis and thermogenesis [6]. Significantly decreased expression of the ADRB3 gene on both the mRNA and protein levels in adipose tissues of obese patients [7] and overweight individuals [8] was observed. Because of its demonstrated functions in lipid metabolism and observed gene expression dysregulation in obesity, ADRB3 could be reasonably expected to constitute a potential pharmacologic target for obesity treatment. The *ADRB3* rs4994 polymorphism (Trp64Arg), a T to C switch leading to the replacement of tryptophan by arginine at position 64, has been related to lower resting metabolic rate [9], weaker response to obesity treatment [10], and increased capacity to gain weight [11] according to scattered evidence. These associations can be imputed to less efficient couple with the G stimulating protein [12] and consequently impaired lipolytic activities [13] led by the rs4994 polymorphism. Given the significance of this *ADRB3* polymorphism, it is necessary to quantitatively assess the strength of its relationship with overweight/obesity risk.

To date, many epidemiological assessments were completed to quantitatively determine the association between the *ADRB3* rs4994 polymorphism and risk of childhood and adolescent overweight/obesity. However, the scattered reports remain inconclusive and did not reach a consensus [14–32]. These studies were plagued by multiple methodological shortcomings, including inadequate statistical power caused by a relatively small sample size, high potential risk of sampling bias, and inconsistent analysis strategies. The aim of this meta-analysis is to quantify more accurately the strength of the genotypic impact of the *ADRB3* rs4994 polymorphism.

## Materials and methods

### Literature and search strategy

A comprehensive literature search for relevant studies published on pre-reviewed journals in five databases [PubMed (https://www.ncbi.nlm.nih.gov/pubmed), Web of Science (https://www.webofknowledge.com/), Cochrane Library (https://www.cochranelibrary.com), China Academic Journals full-text database (CNKI, http://www.cnki.net), and Wanfang data (http://www.wanfangdata.com.cn)] from each database’s inception to 30 May 2019, in Chinese and English, was conducted. Terms for the ADRB3 gene and rs4994 polymorphism (‘Adrenoceptor Beta 3’, ‘ADRB3’, ‘Trp64Arg’, or ‘rs4994’), definitions for single nucleotide polymorphism (‘polymorphism’, ‘polymorphisms’, ‘SNP’, or ‘variant’), synonyms for obesity (‘obesity’, ‘obese’, ‘overweight’, ‘hyperadiposity’, ‘hyperadiposis’, or ‘fatness’), and keywords for ‘childhood and adolescent’ (‘children’, ‘adolescents’, ‘childhood’, or ‘adolescence’) were merged in the Boolean expression for database query.

### Eligibility criteria

The eligibility of individual studies was evaluated by four investigators (YZ, JR, CX and WH) who independently used the predefined exclusion/inclusion criteria. Case–control studies providing original genotype data comparing the allele frequency difference between obese case and control samples were selected. All included studies had to meet the following criteria: (1) Language: studies published in Chinese or English; (2) Participants: children or adolescents; (3) Exposure of interest: genotypes of the *ADRB3* rs4994 polymorphism; (4) Outcomes: obese or overweight. For publications with the same case-control population, only the largest or most complete study was included. Studies with the overall sample size less than 50 were excluded to avoid obvious sampling bias distorting the results.

### Data extraction

A predefined data collection form was used for this review. The extracted data covered information regarding sample characteristics and features of study design. Author’s name, publication year, country, region, polymorphism detection method, definition method for obesity/overweight, and genotype data for case and control were recorded. The Newcastle–Ottawa Scale (NOS) quantification system (http://www.ohri.ca/programs/clinical_epidemiology/oxford.asp, accessed on 1 June 2019) was used to comprehensively assess the quality of included studies in this meta-analysis. The summarized scores for all subscale point items were used to categorize study quality as either low (<4), medium (4–6), or high (>6). Deviations from Hardy–Weinberg equilibrium (HWE) in control populations were assessed with an online calculator (http://ihg.gsf.de/cgi-bin/hw/hwa1.pl). *P*-values less than 0.05 indicate statistical significance for HWE deviation.

### Statistical analysis

All statistical analyses were performed using STATA Statistical Software (Version 14.2; StataCorp LP, College Station, TX, USA). Initially, significance for heterogeneity between studies was evaluated using *Chi*-square based test (a *P* < 0.10 defines the statistical significance). If no significance was detected, a fixed effect model (the Mantel-Haenszel method) would be used [33]. Otherwise, random effects model (the DerSimonian–Laird method) would be applied [34]. Odds ratio (OR), 95% confidence intervals (CI) and the weight for each included study individually or in combination under fixed effect model and random effects model were calculated, estimating the single and pooled effect under homozygote model (CC versus TT), heterozygote model (TC versus TT), dominant model (CC+TC versus TT), recessive model (CC versus TT+TC), and allele model (C versus T). If *P*-value <0.05, the null hypothesis that there is no genetic impact of the *ADRB3* Trp64Arg polymorphism on the increased risk of childhood and adolescent overweight/obesity could be rejected. Subgroup analyses were performed according to the geographical region (East Asia versus others), HWE status (consistent versus inconsistent and unassessable), sample size (no less than 200 versus less than 200), and sex (male versus female). Publication bias was not only visually measured based on the extent of asymmetry of Begg’s funnel plot, and also statistically evaluated by Egger’s regression test [35] and Begg’s rank test [36] (a *P*-value <0.05 defines the statistical significance). If significant publication bias was detected, the Duval and Tweedie’s rank-based ‘trim-and-fill’ method was applied to impute for potentially missing data sets and adjust the effect of publication bias [37]. Leave-one-out sensitivity analysis was performed to calculate the pooled estimates on each subset of the involved studies obtained by omitting exactly one individual study. All statistical variables were reported rounded to two decimal places.

This meta-analysis was deployed in strict accordance with the Preferred Reporting Items for Systematic Reviews and Meta-Analyses (PRISMA) recommendations [38]. Four investigators (YZ, JR, CX and WH) independently performed the meta-analysis and any divergence of the results of study selection, data extraction, quality assessment, and statistical analysis was resolved by consensus.

## Results

### Characteristics of included studies

**Figure 1.**
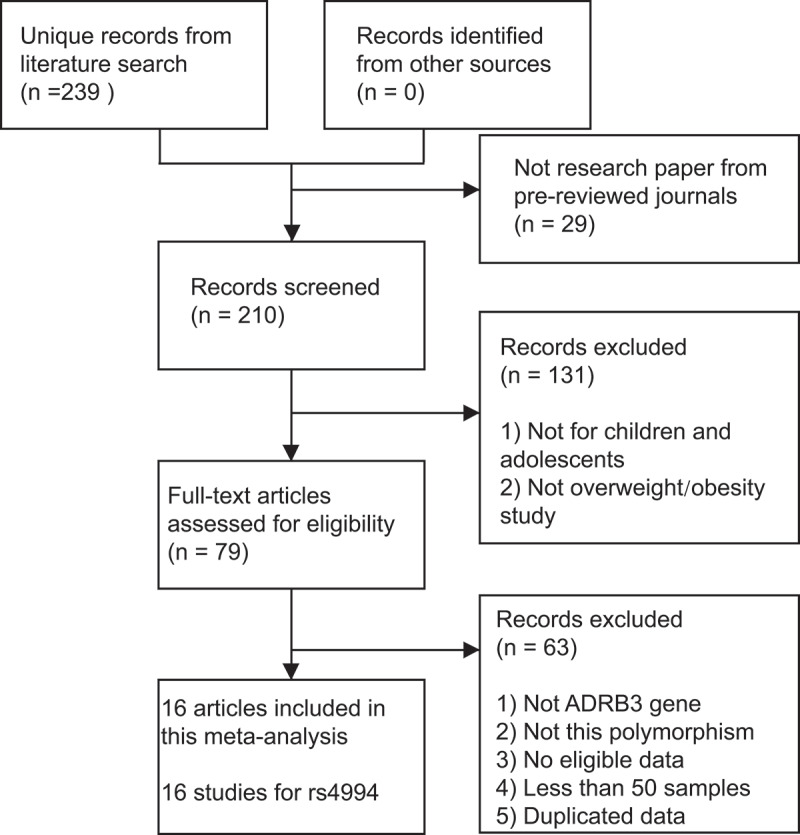
Systematic review flowchart for this meta-analysis

Of the 239 individual publications initially identified, 16 unique studies [14–27,29,30] involving 5,147 overweight/obese cases and 7,350 non-obese controls were ultimately included for further synthetic analyses (Figure 1). Totally, 69.9% of the included subjects came from East Asia. One study for Romanian children was not included because its sample size is less than 50 [28]. Two studies from China were excluded due to duplicated data [21,31,32,31]. The characteristics of the included studies were presented in Table 1. The NOS scores of these studies ranged from 5 to 7, which indicated that all data sets were of high or moderate quality.

**Table 1.** The characteristics of included studies in this meta-analysis

Author	Year	Ref ID	Country	Region	Genotyping method	Obese/Overweight definition	Case	Control	HWE	NOS
Aradillas-Garcia	2017	[29]	Mexico	Latin-America	TaqMan	BMI	348	698	0.23	7
Verdi	2015	[27]	Turkey	West Asia	PCR-RFLP	BMI	130	121	<0.01	5
Kuo	2015	[26]	China	East Asia	TaqMan	BMI	1924	3901	NA	5
Oguri	2013	[24]	Japan	East Asia	PCR-RFLP	BMI	73	59	0.83	5
Zhu	2013	[25]	China	East Asia	PCR-RFLP	BMI	92	71	0.56	5
Csernus	2013	[23]	Hungary	Europe	PCR-RFLP	BMI	703	634	NA	5
Chou	2012	[22]	China	East Asia	TaqMan	BMI	276	277	0.47	6
Peng	2010	[21]	China	East Asia	PCR-RFLP	WHO weight height chart	357	357	0.18	7
Wang	2008	[20]	China	East Asia	PCR-RFLP	BMI	151	85	0.10	5
Zhang	2008	[30]	China	East Asia	PCR-RFLP	Obesity index	95	85	NA	5
Li	2007	[19]	China	East Asia	PCR-RFLP	WHO weight height chart	100	100	0.91	6
Erhardt	2005	[18]	Hungary	Europe	PCR-RFLP	Body weight & body fat content	295	147	0.54	6
Ochoa	2004	[17]	Spain	Europe	PCR-RFLP	BMI	185	185	0.25	5
Mo	2001	[16]	China	East Asia	PCR-RFLP	NA	90	87	NA	6
Endo	2000	[15]	Japan	East Asia	PCR-RFLP	Obesity index	90	463	0.80	5
Hinney	1996	[14]	Germany	Europe	PCR-RFLP	BMI	238	80	0.43	5

HWE: Hardy–Weinberg equilibrium; NOS: Newcastle–Ottawa scale; NA: not available. BMI: body mass index, calculated according to the weight/height^2^ (kg/m^2^) formula. Obesity index: calculated according to the (real weight-standard weight)/standard weight * 100 formula.

### Overall and subgroup meta-analyses

**Figure 2.**
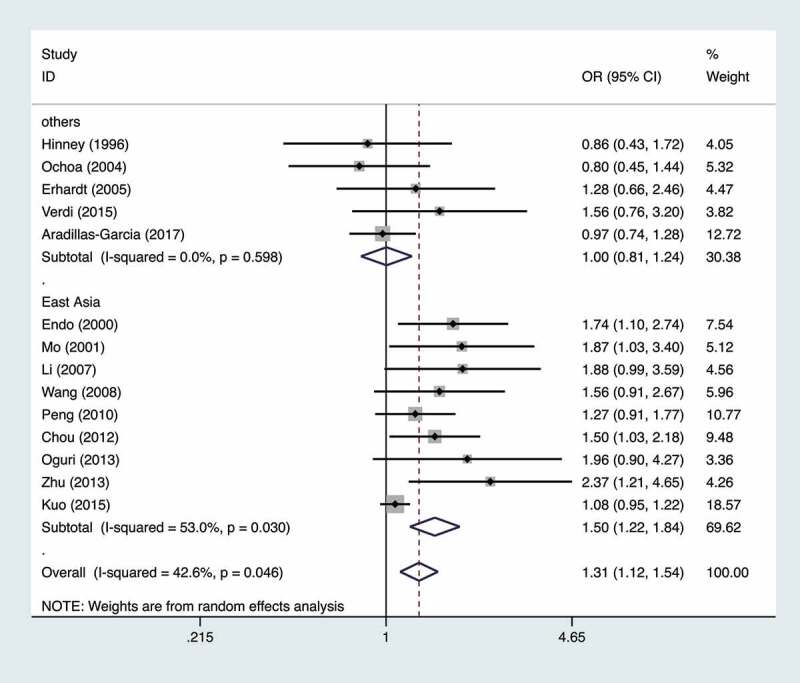
Forest plot showing the association between the *ADRB3* rs4994 polymorphism and risk of childhood and adolescent overweight/obesity. Odds ratio (OR) and 95% confidence interval (95% CI) were calculated under the dominant model. The random effects model was used to assess the pooled estimates. The included studies were stratified according to geographical regions. The grey squares represent the weight of the sample size under the random effects model. The black dot in the square represented the OR for each included study. The black horizontal lines showed the corresponding 95% CI. The black solid vertical line showed the null effect (OR = 1). The red dashed vertical line showed the pooled estimate. The hollow diamonds at the bottom represented the ORs and 95% CIs for the overall population and subgroups. The data sets drawn from ref[23] and ref[30] were not included because they did not provide genotype data for the dominant model

Significant between-study heterogeneity was detected in heterozygote model (*p* = 0.08) and dominant model (*p* = 0.05), so random effects model was used. Fixed effect model was adopted for synthetic analyses in allele model, homozygote model, and recessive model. Pooled estimates of overall and subgroup synthetic analyses were summarized in Table 2. To be brief, significant association between the *ADRB3* rs4994 polymorphism and obese risk was detected in allele model (OR 1.23, 95% CI 1.10–1.38), heterozygote model (OR 1.39, 95% CI 1.16–1.68), and dominant model (OR 1.31, 95% CI 1.12–1.54), but not in homozygote model (OR 1.36, 95% CI 0.90–2.06) and recessive model (OR 1.26, 95% CI 0.83–1.90). Statistical significance for allele model, heterozygote model, and dominant model could be detected in both male and female subgroups. When stratifying the data sets according to the geographical region, we identified a statistically significant association between this polymorphism and obese risk in the subgroup of East Asia in allele model, homozygote model, heterozygote model, and dominant model (Figure 2). However, no statistical significance could be reached in the non-East Asian subgroup in all genetic models. After excluding studies that deviated from HWE, no significant alterations of results were detected, suggesting our pooled estimates were statistically reliable. The significance level remained unchanged in both subgroups for studies with large and small sample size, thus illustrating the robustness of the conclusions. Furthermore, no heterogeneity significance could be identified in neither subgroups of heterozygote model and dominant model, which suggested that the between-study heterogeneity in this meta-analysis was significantly attributed to sample size.

**Table 2.** Results of overall and subgroup analyses for the *ADRB3* rs4994 polymorphism and risk of childhood and adolescent overweight/obesity

	Allele model			Homozygote model			Heterozygote model			Dominant model			Recessive model		
Comparison	OR (95% CI)	*P*	*P_h_*	OR (95% CI)	*P*	*P_h_*	OR (95% CI)	*P*	*P_h_*	OR (95% CI)	*P*	*P_h_*	OR (95% CI)	*P*	*P_h_*
Overall	1.23(1.10,1.38)	<0.01	0.24	1.36(0.90,2.06)	0.15	0.56	1.39(1.16,1.68)	<0.01	0.08	1.31(1.12,1.54)	<0.01	0.05	1.26(0.83,1.90)	0.28	0.64
Region															
East Asia	1.47(1.25,1.71)	<0.01	0.80	1.97(1.09,3.56)	0.02	0.76	1.60(1.34,1.90)	<0.01	0.57	1.50(1.22,1.84)	<0.01	0.03	1.68(0.94,3.00)	0.08	0.73
Others	1.04(0.89,1.22)	0.62	0.74	0.92(0.50,1.69)	0.79	0.54	1.01(0.82,1.26)	0.90	0.46	1.01(0.82,1.24)	0.96	0.60	0.92(0.50,1.68)	0.79	0.51
HWE															
Consistent	1.25(1.11,1.41)	<0.01	0.15	1.45(0.95,2.21)	0.09	0.63	1.28(1.11,1.47)	<0.01	0.12	1.34(1.11,1.62)	<0.01	0.09	1.34(0.88,2.04)	0.18	0.71
Others*	1.17(0.90,2.52)	0.25	0.76	0.33(0.03,3.23)	0.34	NA	2.16(1.32,3.53)	<0.01	0.66	1.11(0.99,1.25)	0.08	0.14	0.30(0.03,2.97)	0.31	NA
Sample Size															
<200	1.82(1.16,2.84)	<0.01	0.84	1.27(0.30,5.32)	0.74	0.13	2.30(1.54,3.45)	<0.01	0.76	2.05(1.39,3.02)	<0.01	0.87	1.02(0.25,4.17)	0.98	0.10
≥200	1.20(1.07,1.35)	<0.01	0.30	1.37(0.89,2.11)	0.16	0.61	1.24(1.07,1.43)	<0.01	0.27	1.15(1.05,1.26)	<0.01	0.16	1.28(0.83,1.97)	0.26	0.74
Sex															
male	1.31(1.03,1.67)	0.03	0.75	1.45(0.46,4.54)	0.53	0.93	1.38(1.05,1.83)	0.02	0.56	1.44(1.12,1.85)	<0.01	0.54	1.35(0.43,4.21)	0.61	0.91
female	1.74(1.29,2.35)	<0.01	0.63	2.47(0.54,11.18)	0.24	0.83	1.90(1.36,2.66)	<0.01	0.39	2.01(1.48,2.73)	<0.01	0.45	1.98(0.44,8.97)	0.37	0.81

HWE: Hardy–Weinberg equilibrium; OR: odds ratio; 95% CI: 95% confidence interval; NA: not available. * Studies inconsistent with HWE and unassessable were classified into subgroup ‘Others’.

### Publication bias analysis

**Figure 3.**
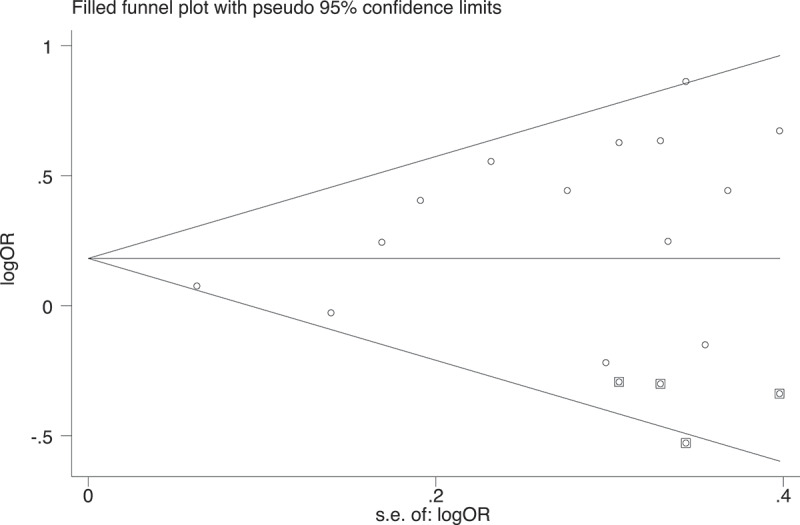
Filled funnel plot with imputed studies under the dominant model. Using the ‘trim-and-fill’ method, the pooled estimates are adjusted for possible missing data sets (squared circles) amongst published studies (hollow circles). The log odds ratio (OR) stands for the natural logarithm transferred OR of individual data sets. The standard error of the log OR represents the standard error of the natural logarithm transferred OR of individual data sets. The data sets drawn from ref[23] and ref[30] were not included because they did not provide genotype data for the dominant model

The degree of publication bias was estimated based on the asymmetry of the funnel plots (data not shown). Begg’s rank correlation test for publication bias showed no statistical significance in all five genetic models (allele model: *p* = 0.36, homozygote model: *p* = 0.72, heterozygote model: *p* = 0.20, dominant model: *p* = 0.19, recessive model: *p* = 0.72). However, Egger’s regression test revealed there is slightly significant publication bias in the dominant model (*p* = 0.02), but not in the other models (allele model: *p* = 0.18, homozygote model: *p* = 0.57, heterozygote model: *p* = 0.10, recessive model: *p* = 0.71). The summary analysis incorporating four additional hypothetical studies using the trim-and-fill method continued to reveal a statistically significant association between the *ADRB3* rs4994 polymorphism and risk of childhood and adolescent overweight/obesity (OR 1.20, 95% CI 1.03–1.40, Figure 3), suggesting the robustness of the pooled estimates from the dominant model.

### Sensitivity analysis

**Figure 4.**
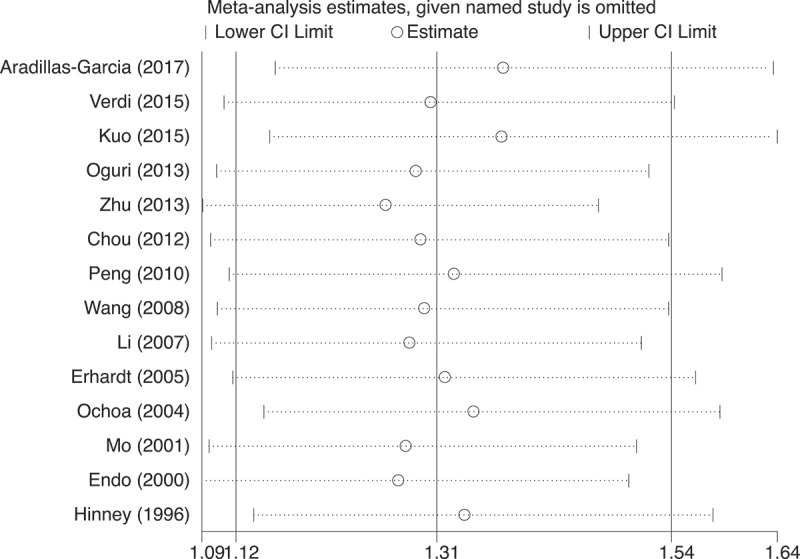
Sensitivity analysis for the pooled estimates under the dominant model. For each omitted data set listed on the left, summary statistics for the resulting pooled estimates are presented as odds ratio (OR, hollow circle) with 95% confidence interval (CI, horizontal line). The random effects model was used to assess pooled estimates. The data sets drawn from ref[23] and ref[30] were not included because they did not provide genotype data for the dominant model

The leave-one-out sensitivity analysis iteratively removed one data set at a time to determine whether the statistical significance of the pooled estimates was driven by any single study. No statistically significant changes could be observed (Figure 4), suggesting the stability of the results.

## Discussion

There is still uncertainty regarding the genetic impact of the *ADRB3* rs4994 polymorphism on the risk of childhood and adolescent overweight/obesity despite previous case–control studies [14–32]. Our systematic literature review and meta-analysis identified statistical significance under allele model, heterozygote model, and dominant model, which suggest that the C allele of this polymorphism could be a risk factor. Subgroup analysis by sex showed that statistical significance for allele model, heterozygote model, and dominant model could be detected in both male and female subgroups. However, given the limited number of included studies and sample size, these significances would not conclude whether or not gender could influence the impact of this polymorphism. Further stratified analyses according to geographical region revealed significantly higher risk of overweight/obesity in the East Asia subgroup under allele model, homozygote model, heterozygote model, and dominant model, suggesting the possibility that there may be some interaction effects with other genetic variables with ethnicity or regional specificity. The variances of the lifestyle should also be taken into account.

Meta-analyses pooling scattered epidemiological studies are generally prone to between-study heterogeneity and bias, which could partly impact the difficulty in drawing conclusions based on pooled estimates [39]. In the heterozygote model and dominant model, substantial heterogeneity with regard to the overall population was detected. The conclusion of individual study with small sample size may be undermined by methodological weaknesses such as failure to control potential confounding factors, which could lead to significant between-study heterogeneity in meta-analysis. Stratified analyses according to sample size remarkably reduced the significance level of heterogeneity in this study. However, the conclusions based on the pooled estimates in both subgroups remained stable. This supported the robustness of our meta-analysis. In addition, the results of the leave-one-out sensitivity analysis ensured that no single data set could dominate the statistical significance of the pooled estimates, indicating the validity of our conclusions. Although statistical significance for publication bias was detected in the dominant model, the trim-and-fill method adjusting for asymmetry of funnel plot confirmed that a positive association between this polymorphism and overweight/obesity risk is unlikely to be due to publication bias, suggesting the authenticity of our results. In brief, these above aspects could be regarded as the manifestation of concrete reliability for this meta-analysis.

This meta-analysis has several merits. Our study endorsed the recommendations by the PRISMA guidelines [38]. The broad-scope search of multiple literature databases, stringent study selection and data extraction, standardized statistical analysis processes, and rigorous interpretation of final results substantially reinforced our confidence in the validity of this study. However, a clear understanding of the drawbacks inherent in our approach should be reached to disclose the limitations of the results. First of all, the primary limitation of this meta-analysis is the differing definitions of overweight/obesity across ethnicities and countries. Although the heterogeneity is not unexpected given methodological nature of the included studies, this complicating factor does impact the ability to precisely calculate the pooled estimates. Furthermore, most of the included studies were conducted in East Asia, which significantly impacted the ethnic diversity and could lead to sampling bias. Moreover, this quantitative synthesis only assessed the genetic impact of the rs4994 polymorphism. It is still unable to exclude the likelihood that other functional polymorphisms of the ADRB3 gene also contribute to the risk of overweight/obesity. In addition, besides the individual contribution of each polymorphism, whether the combined polymorphisms, so-called haplotypes, could exert a synergistic effect has yet to be answered. Last but not least, confounding factors including over- or underdiagnosis, concomitant diseases, error in the genotyping assay, environmental exposure, socioeconomic status, and behaviour and lifestyle habits of the family should also be taken into account in future studies. Thus, the results of this meta-analysis should be interpreted with caution.

In conclusion, the results of this meta-analysis evaluating the genetic impact of the *ADRB3* rs4994 polymorphism on the risk of overweight/obesity among children and adolescents are reassuring. There is a statistically significant association between the rs4994 polymorphism and overweight/obesity risk. However, this meta-analysis shows that the C allele of this polymorphism in the *ADRB3* gene is a risk factor for overweight/obesity only in children and adolescents from East Asia.

## References

[1] Cataldo R, Huang J, Calixte R, et al. Effects of overweight and obesity on motor and mental development in infants and toddlers. Pediatr Obes. 2016;11(5):389–396. 26487592 10.1111/ijpo.12077

[2] Pollock NK. Childhood obesity, bone development, and cardiometabolic risk factors. Mol Cell Endocrinol. 2015;410:52–63. 25817542 10.1016/j.mce.2015.03.016 PMC4444415

[3] Wagner IV, Sabin MA, Pfäffle RW, et al. Effects of obesity on human sexual development. Nat Rev Endocrinol. 2012;8(4):246. 22290357 10.1038/nrendo.2011.241

[4] Mirza NM, Yanovski JA. Prevalence and consequences of pediatric obesity. In: Bray GA, Bouchard C, editors. Handbook of obesity: epidemiology, etiology, and physiopathology. Boca Raton, FL: CRC Press; 2014. p. 55–74.

[5] Robidoux J, Martin TL, Collins S. Beta-adrenergic receptors and regulation of energy expenditure: a family affair. Annu Rev Pharmacol Toxicol. 2004;44:297–323. 14744248 10.1146/annurev.pharmtox.44.101802.121659

[6] Collins S, Surwit RS. The beta-adrenergic receptors and the control of adipose tissue metabolism and thermogenesis. Recent Prog Horm Res. 2001;56:309–328. 11237219 10.1210/rp.56.1.309

[7] Kurylowicz A, Jonas M, Lisik W, et al. Obesity is associated with a decrease in expression but not with the hypermethylation of thermogenesis-related genes in adipose tissues. J Transl Med. 2015;13(1):31. 25622596 10.1186/s12967-015-0395-2 PMC4314800

[8] Cao W-Y, Liu Z, Guo F, et al. Adipocyte ADRB3 down-regulated in Chinese overweight individuals adipocyte ADRB3 in overweight. Obes Facts. 2018;11(6):524–533. 30580338 10.1159/000495116 PMC6341365

[9] Walston J, Silver K, Bogardus C, et al. Time of onset of non-insulin-dependent diabetes mellitus and genetic variation in the beta 3-adrenergic-receptor gene. N Engl J Med. 1995;333(6):343–347. 7609750 10.1056/NEJM199508103330603

[10] Yoshida T, Sakane N, Umekawa T, et al. Mutation of beta 3-adrenergic-receptor gene and response to treatment of obesity. Lancet. 1995;346(8987):1433–1434. 10.1016/s0140-6736(95)92452-3 7475854

[11] Clement K, Vaisse C, Manning BS, et al. Genetic variation in the beta 3-adrenergic receptor and an increased capacity to gain weight in patients with morbid obesity. N Engl J Med. 1995;333(6):352–354. 7609752 10.1056/NEJM199508103330605

[12] Piétri‐Rouxel F, St John Manning B, Gros J, et al. The biochemical effect of the naturally occurring Trp644→ Arg mutation on human β3‐adrenoceptor activity. Eur J Biochem. 1997;247(3):1174–1179. 9288945 10.1111/j.1432-1033.1997.01174.x

[13] Umekawa T, Yoshida T, Sakane N, et al. Trp64Arg mutation of beta3-adrenoceptor gene deteriorates lipolysis induced by beta3-adrenoceptor agonist in human omental adipocytes. Diabetes. 1999;48(1):117–120. 9892231 10.2337/diabetes.48.1.117

[14] Hinney A, Lentes K, Rosenkranz K, et al. β 3-adrenergic-receptor allele distributions in children, adolescents and young adults with obesity, underweight or anorexia nervosa. Int J Obesity. 1997;21(3):224. 10.1038/sj.ijo.0800391 9080262

[15] Endo K, Yanagi H, Hirano C, et al. Association of Trp64Arg polymorphism of the β 3-adrenergic receptor gene and no association of Gln223Arg polymorphism of the leptin receptor gene in Japanese schoolchildren with obesity. Int J Obesity. 2000;24(4):443. 10.1038/sj.ijo.0801177 10805501

[16] Mo B, Chen R, Guo X, et al. The role of β 3-adrenergic receptor Trp/Arg mutation in childhood obesity. Chin J Med Genet. 2001;18(5):371–374. 11592045

[17] Ochoa M, Marti A, Azcona C, et al. Gene–gene interaction between PPARγ2 and ADRβ3 increases obesity risk in children and adolescents. Int J Obesity. 2004;28(S3):S37. 10.1038/sj.ijo.0802803 15543217

[18] Erhardt É, Czakó M, Csernus K, et al. The frequency of Trp64Arg polymorphism of the β 3-adrenergic receptor gene in healthy and obese Hungarian children and its association with cardiovascular risk factors. Eur J Clin Nutr. 2005;59(8):955. 15942638 10.1038/sj.ejcn.1602164

[19] Li Q, Xu J, Zhang Y, et al. Effects of variation of the β3-adrenergic-receptor and uncoupling Protein-2 gene polymorphism on children simple obesity. Chin J Sch Health. 2007;28(3):249–252.

[20] Wang Z, Gao W-H. Gene polymorphism study of β3 adrenergic receptor in school aged children in Shenzhen. Chin J Misdiagnostics. 2008;8(25):6052–6053.

[21] Peng A, Yang S, Zhang B, et al. Detection and significance of Trp64Arg Mutat ion of β3-AR in preschool children with simple obesity. Acta Medicinae Universitatis Scientiae Et Technologiae Huazhong. 2010;39(6):868–871.

[22] Chou YC, Tsai CN, Lee YS, et al. Association of adrenergic receptor gene polymorphisms with adolescent obesity in Taiwan. Pediatr Int. 2012;54(1):111–116. 22115535 10.1111/j.1442-200X.2011.03516.x

[23] Csernus K, Pauler G, Erhardt E, et al. Uncoupling protein‐2 gene polymorphisms are associated with obesity in Hungarian children. Acta Paediatrica. 2013;102(5):e200–e04. 23432701 10.1111/apa.12181

[24] Oguri K, Tachi T, Matsuoka T. Visceral fat accumulation and metabolic syndrome in children: the impact of T rp64 A rg polymorphism of the beta3‐adrenergic receptor gene. Acta Paediatrica. 2013;102(6):613–619. 23282015 10.1111/apa.12149

[25] Zhu C, Zhang J, Xu P, et al. Association of polymorphism of β3-adrenergic receptor gene and the leptin gene with Kazak obese children in Xinjiang. Chin J Appl Clin Pediatr. 2013;28(11):816–819.

[26] Kuo N-W, Tung K-Y, Tsai C-H, et al. β3-Adrenergic receptor gene modifies the association between childhood obesity and asthma. J Allergy Clin Immunol. 2014;134(3):731–33. e3. 24786241 10.1016/j.jaci.2014.03.018

[27] Verdi H, Kınık ST, Yalçın YY, et al. β-3AR W64R polymorphism and 30-Minute post-challenge plasma glucose levels in obese children. J Clin Res Pediatr Endocrinol. 2015;7(1):7. 25800470 10.4274/jcrpe.1629 PMC4439896

[28] Ursu R-I, Cucu N, Ursu G-F, et al. Frequency study of the FTO and ADRB3 genotypes in a Romanian cohort of obese children. Rom Biotechnol Lett. 2016;21(3):11611.

[29] Aradillas-Garcia C, Cruz M, Pérez-Luque E, et al. Obesity is associated with the Arg389Gly ADRB1 but not with the Trp64Arg ADRB3 polymorphism in children from San Luis Potosí and León, México. J Biomed Res. 2017;31(1):40. 10.7555/JBR.30.20150169 PMC5274511 28808184

[30] Zhang J, Li M, Xu P, et al. Distribution of beta3-adrenergic receptor Trp64Arg mutation of Karzak school-aged children in Xinjiang. J Xinjiang Med Univ. 2008;31(1):74–76.

[31] Mo B, Chen R, Guo X, et al. The detection and significance of Trp64Arg Mutation of β3-adrenergic receptor in school-age children. Acta Universitatis Medicinalis Nanjing. 2000;20(9):359–361.

[32] Peng A, Yang S, Zhang B, et al. Detection and significance of Trp64Arg mutation of β3-AR and temperament in preschool children with simple obesity. Chin J Sch Health. 2011;32(2):186–187.

[33] Mantel N, Haenszel W. Statistical aspects of the analysis of data from retrospective studies of disease. J Natl Cancer Inst. 1959;22(4):719–748. 13655060

[34] DerSimonian R, Laird N. Meta-analysis in clinical trials. Control Clin Trials. 1986;7(3):177–188. 3802833 10.1016/0197-2456(86)90046-2

[35] Egger M, Smith GD, Schneider M, et al. Bias in meta-analysis detected by a simple, graphical test. BMJ. 1997;315(7109):629–634. 9310563 10.1136/bmj.315.7109.629 PMC2127453

[36] Begg CB, Mazumdar M. Operating characteristics of a rank correlation test for publication bias. Biometrics. 1994;1088–1101. DOI:10.2307/2533446 7786990

[37] Duval S, Tweedie R. Trim and fill: a simple funnel‐plot–based method of testing and adjusting for publication bias in meta‐analysis. Biometrics. 2000;56(2):455–463. 10877304 10.1111/j.0006-341x.2000.00455.x

[38] Moher D, Liberati A, Tetzlaff J, et al. Preferred reporting items for systematic reviews and meta-analyses: the PRISMA statement. BMJ. 2009;339. DOI:10.1136/bmj.b2535. PMC3090117 21603045

[39] Higgins JPT, Thompson SG. Quantifying heterogeneity in a meta-analysis. Stat Med. 2002;21(11):1539–1558. 12111919 10.1002/sim.1186

